# Long-term Survival Rate Following Myocardial Infarction and the Effect of Discharge Medications on the Survival Rate

**DOI:** 10.34172/jrhs.2022.102

**Published:** 2022-12-29

**Authors:** Sahar Bayat, Seyed Saeed Hashemi Nazari, Yadollah Mehrabi, Mohammad Sistanizad

**Affiliations:** ^1^Prevention of Cardiovascular Disease Research Center, Department of Epidemiology, School of Public Health and Safety, Shahid Beheshti University of Medical Sciences, Tehran, Iran; ^2^Safety Promotion and Injury Prevention Research Center, Department of Epidemiology, School of Public Health and Safety, Shahid Beheshti University of Medical Sciences, Tehran, Iran; ^3^Department of Clinical Pharmacy, Faculty of Pharmacy, Shahid Beheshti University of Medical Sciences, Tehran, Iran

**Keywords:** Acute myocardial infarction, Cox proportional-hazards model, Survival rate

## Abstract

**Background:** The evaluation of the risk factors associated with the long-term survival rate of patients with myocardial infarction (MI) and the effects of discharge medications can significantly help select the most effective strategies for improving treatment.

**Study Design:** A retrospective cohort study.

**Methods:** The participants of this retrospective cohort study were 21,181 patients who suffered from MI and were hospitalized in the cardiac care unit (CCU) of different public, private, and military hospitals in Iran from 20 March 2013 to 20 March 2014. Participants were followed up until February 2020 for any cardiovascular disease (CVD) mortality. To evaluate survival rate, the differences between groups, and the factors related to MI death, Kaplan-Meier, log-rank test, and Cox proportional-hazards model were used, respectively.

**Results:** One, three, five, and seven-year survival rates of patients were 88%, 81%, 78%, and 74%, respectively. Regarding the interaction effect of prescribed medical drugs, the highest 7-year survival rate of 86% (95% CI: 72%, 93%) was related to people who consumed anticoagulants, aspirin, clopidogrel, beta blockers, angiotensin-converting enzymes (ACEs), and angiotensin II receptor antagonist simultaneously. Considering the effect of other variables, the consumption of anticoagulants was associated with a decrease in survival rate (HR=1.13 CI: 1.06, 1.19).

**Conclusion:** As evidenced by the results of this study, different combinations of prescribed medication drugs had protective effects on long-term mortality compared to the group without any drug. Nonetheless, according to the drugs in each combination therapy, this protective effect ranged from HR=0.27 to HR=0.89. It is recommended that further studies compare the long-term effects of different drug combinations and also consider adherence to treatment in evaluating the effects of these combinations.

## Background

 Cardiovascular diseases (CVDs) are the first leading cause of death worldwide. A total of 17.9 million people die annually due to CVDs, one-third of whom are under 70.^1^ This equals 330 million years of life lost due to the premature death of 35.6 million years of life with disabilities.^2^ Despite rapid diagnostic methods and therapeutic progress, one-third of patients who suffer from myocardial infarction (MI) will die, and two-thirds of those who survive never fully recover and return to normal life. These diseases impose a high cost on the health care system of countries.^3^ According to the World Health Organization, about 23.6 million people will die by 2030 due to CVDs.^4^

 The clinical symptoms of cardiac ischemia include chest pain, upper body pain, jaw pain, or epigastric discomfort caused by pressure or stress. The pain caused by acute MI takes long for at least 20 minutes. The discomfort is usually diffusing, not localized, nor positional, or affected by the movement of the region and may be accompanied by asthma, sweating, nausea, or syncope. These symptoms are not specific to myocardial ischemia and can be detected incorrectly and, therefore, can be attributed to digestive, nervous, lung, or muscle disorders. Infarction may be accompanied by atypical symptoms or even without signs.^5^

 The risk factors of heart stroke are divided into two types: fixed and changeable. Fixed factors include individuals with a family background of CVD, people who have at least one family member with hypertension, type 2 diabetes, high cholesterol, high low-density lipoprotein (LDL), and low high-density lipoprotein (HDL), those over 60 years of age and above, and women after menopause. On the other hand, changeable risk factors include hypertension, smoking (active and second-hand smoke), abnormal obesity, hyperlipidemia, diabetes plus stress, low fruit and vegetable consumption, and low physical activity. Low physical activity is the leading cause of death, responsible for more than 90% of MIs.^6,7^

 A combination of these adverse risk factors can affect long-term survival rates.^8^ Among the drugs used in the treatment of CVD, at least one of the statins and calcium channel blockers is prescribed in more than half of the patients. Other drugs, such as angiotensin-converting enzyme (ACE) inhibitors, alpha-blockers, angiotensin II receptor blockers, antiplatelet therapy (including aspirin), glycosides, centrally acting antihypertensives, fibrates, other lipid-lowering drugs, loop diuretics, nicorandil, nitrates, superventricular antiarrhythmic, and beta-blockers are also prescribed.^9^

 Survival is an important outcome of acute MI. The follow-up period is short in most survival studies. Very few studies, for instance, have assessed five-year periods. Studies of long-time periods are of paramount importance since they may exhibit the influence of factors that affect their survival differently from short periods. The premature death of 30 days after acute MI is about 30%.^10^ Various studies have reported a five-year survival rate, ranging from 48.2%-62.3%.^11,12^

 Reliable survival estimates are important to investigate any long-term condition at the population level to monitor trends in the prognosis and allocate appropriate services. At the patient level, these estimates allow informed discussions and shared decision-making about treatment options, as well as advanced care planning, and take appropriate measures to increase the lifespan and survival of patients. In light of the aforementioned issues, the present study aimed to estimate the long-term survival rate and its associated risk factors, as well as the effect of prescribed drugs at the time of hospital discharge on long-term survival following MI.

## Methods

 We performed a retrospective cohort study using data from theMI registry system from 20 March 2013 to 20 March 2014. The participants of this study were 21 181 patients who suffered from MI and were hospitalized in the cardiac care unit (CCU) of different public, private, and military hospitals in Iran. Their information was extracted from the MI registry system of the country, and they were followed up to February 2020 for each mortality (ICD10 codes I00-I09, I11, I13, I20-I51, I21, I22, I24, I250, I33, I30- I31, I40, I50, I26-I28, I34-I38, I42-I49, I51) caused by CVD. In this registry system, all medical information of MI patients during hospitalization is registered based on the International Classification of Diseases (ICD-10) coding system, including;

1. Acute subendocardial MI
2. Acute transmural MI of other sites
3. Acute transmural MI of inferior
4. Acute transmural MI of the anterior wall
5. Acute MI of unspecified site
6. Acute transmural MI of the anterior wall and inferior wall
7. Acute transmural MI of anterior wall and other sites
8. Acute transmural MI of the inferior wall and other sites
9. Acute MI with non-ST elevation with location
10. Acute transmural MI of the anterior wall, inferior wall, and other sites

The study included patients suffering from acute MI whose data have been recorded in the MI registry system. Patients who could not be followed through the registration system and those with missing or invalid IDs were excluded from the study. Deaths due to reasons other than CVDs and people who were alive at the end of the study were considered censored. The studied variables included demographic characteristics, location of MI according to ICD10 categorization, heart disease risk factors (coronary diseases, hypertension, diabetes, smoking, and hyperlipidemia), clinical symptoms at the time of MI, presence of arrhythmia and its different types, post-MI complications, the history of receiving percutaneous coronary intervention (PCI)/coronary artery bypass grafting (CABG) treatments, and receiving drug groups at the time of discharge (diuretic, anticoagulant, aspirin, clopidogrel, nitrate agent, calcium channel blocking agent, beta blocking agent, statin prophylaxis, angiotensin converting enzyme, and angiotensin II receptor antagonist). The prescribed medications were categorized into eight groups (Table 1) with 55 combinations.

**Table 1:** Drug groups when discharging patients with acute myocardial infarction

**Group 1**	**Group 2**	**Group 3**	**Group 4**	**Group 5**	**Group 6**	**Group 7**	**Group 8**
1. Diuretic	1. Anticoagulant	1. Aspirin 2. Clopidogrel	1. Nitrate agent	1. Calcium channel blocking agent	1. Beta blocking agent	1. Statin prophylaxis	1. Angiotensin-converting enzyme 2. Angiotensin II receptor antagonist

###  Follow up

 The main outcome of the study was death due to CVD. People whose first MI was recorded in the MI-Registry system were included in the study, eliminating those with secondary MI or more. The follow-up of those who had MI was performed through a cross-match of their national code with the health system death registry data. The time between the incidence of acute MI and death due to CVD was considered the survival time. Survival times for those who had died due to other causes were computed between MI and the date of death and considered censored. People who were alive at the end of the study were regarded as censored, and their survival time was calculated by differencing the MI date and a month before the last follow-up. The registration of death in the civil registry is delayed by one month. This study was approved by the Ethics Committee of the School of Public Health and Safety, Shahid Beheshti University of Medical Sciences (IR.SBMU.PHNS.REC.1399.135).

###  Statistical Analyses

 Continuous variables were reported as mean and standard deviations, while the categorical variables were presented as counts and percentages. Kaplan-Meier method was used to calculate survival rate at the levels of gender, age groups, patients’ medical history, the occurrence of heart complications, location of MI, and prescripted drugs at the time of discharge. Differences between the groups were checked using the Log-rank test. Cox proportional-hazards model was utilized To investigate the factors related to death due to MI and controlling probable confounders.

 Crude mortality rate and cause-specific mortality rate are elaborated. Crude mortality is defined as the total number of deaths during a seven-year period divided by the number of people with MI over a period of seven years. The cause-specific mortality rate is determined by the number of deaths due to CVDs divided by the number of people with MI during the seven-year study period. In the current study, there was an underestimate since the deaths due to MI before reaching the hospital and deaths at home were not registered.

 The univariate Cox model was carried out, and the impact of gender, level of education, coronary diseases, diabetes, hypertension, hyperlipidemia, history of PCI/CABG treatment, having clinical symptoms at the time of MI, post-MI complications, arrhythmia, location of MI, and received medication types at the time of discharge were measured. Thereafter, the variables with p-value less than 0.2 in univariate were entered into the multivariate analysis. We tested the proportional hazard assumption by plotting Schoenfeld residuals over time. Trends over time were evident for diabetes, hyperlipidemia, smoking, and post-MI heart complications in the model. Following that, the effect of the interaction of these variables with time entered into the model, and their hazard ratio was reported.

 To control the confounding effect of age, the date of birth was considered the origin of time, and the date of hospitalization was regarded as the beginning of the study. The analyses were carried out using Stata software (version 14), and all analyses were reported with a significant level of less than 0.05 and a 95% confidence interval.

## Results

 Out of the patients registered in the country’s registration system and follow-up to the end of 2020, 5452 cases died of CVD. A total of 1184 subjects died of non-cardiovascular causes, and 14 545 people were alive by the end of the study. The mean age of patients was 62.10 ± 13.41(a minimum of 4 and a maximum of 103 years old). In terms of gender, the majority of patients (72.37%) were male. Most patients were in the age group of 50-60 years, and 46.45% of them were illiterate. Hypertension (36.22%) was the most common risk factor among patients, and hyperlipidemia had the lowest frequency.

 The most common location of MI was acute transmural MI of the anterior wall (31.86%.). The most frequently prescribed drug was group 3 (aspirin/clopidogrel), with an absolute frequency of 19 929, and the least frequently administrated drug was group 6 (calcium channel blockers) with an absolute frequency of 1583. Gender-stratified survival rates of the patients are shown in Table 2. One-year survival rate was 88% (95% CI: 87%, 88%), three-year 81% (95% CI: 80%, 82%), five-year 78% (95% CI: 77%, 78%), and 7-year survival rate was 74% (95% CI: 73%, 75%). The survival rate in women was significantly lower than in men (*P* < 0.001). The seven-year crude mortality rate was 31.32%, and the cause-specific mortality rate was 25.74%.

**Table 2:** Survival rates by gender groups

**Gender**	**N**	**Crude mortality**	**Cause-specific mortality**	**1-Year survival ** **(95% CI)**	**3-Year survival** **(95% CI)**	**5-Year survival** **(95% CI)**	**7-Year survival** **(95% CI)**
Male	5853	27.73%	22.48%	0.90 (0.89, 0.90)	0.84 (0.83,0.84)	0.81 (0.80, 0.81)	0.77 (0.77, 0.78)
Female	15328	40.74%	34.27%	0.82 (0.81, 0.83)	0.73 (0.73, 0.75)	0.70 (0.69, 0.71)	0.66 (0.64, 0.67)
Total	21181	31/32%	25.74%	0.88 (0.87, 0.88)	0.81 (0.80, 0.82)	0.78 (0.77, 0.78)	0.74 (0.73, 0.75)

Table 3 displays the survival rate for different medication groups. In this table, the survival rates and adjusted hazard ratios are calculated for each medication group. The comparison group for each medication group was the patients who had not received that drug group; moreover, as illustrated, people who took groups 1 and 5 had the lowest survival rate compared to other drug groups (73% CI: 72%, 75% and 73% CI: 71%, 75%). Survival rates in people who received group 6 drugs were higher than in other medication groups. There is a statistically significant difference between survival rates in people with different drug groups (*P* < 0.001).

**Table 3:** Risk factors associated with death from acute myocardial infarction based on the Cox multivariate model

**Drug groups received at discharge**	**N**	**Survival Rate (95% CI)**				**Crude hazard ratio**		**Adjusted hazard ratio **^a^	
		**1-Year **	**3-Year **	**5-Year **	**7-Year**	**HR (95% CI)**	***P*** ** value**	**HR (95% CI)**	***P*** ** value**
Diuretic	3175	0.87 (0.86,0.88)	0.80 (0.79,0.81)	0.77 (0.75,0.78)	0.73 (0.72,0.75)	1.04 (0.96,1.12)	0.310	1.03 (0.96,1.11)	0.350
Anticoagulant	12 710	0.88 (0.87,0.88)	0.81 (0.81,0.82)	0.78 (0.77,0.78)	0.74 (0.73,0.75)	1.00 (0.95,1.06)	0.230	1.13 (1.06,1.19)	0.001
Aspirin, clopidogrel	19 929	0.88 (0.88,0.89)	0.82 (0.81,0.82)	0.78 (0.78,0.79)	0.74 (0.74,0.75)	0.82 (0.78,0.86)	0.001	0.64 (0.56,0.73)	0.001
Nitrate agent	17 947	0.88 (0.88,0.89)	0.82 (0.81,0.82)	0.78 (0.78,0.79)	0.75 (0.74,0.75)	0.80 (0.74,0.85)	0.001	0.93 (0.86,1.01)	0.120
Calcium channel blocking agent	1583	0.87 (0.85,0.89)	0.81 (0.79,0.83)	0.77 (0.75,0.79)	0.73 (0.71,0.75)	0.96 (0.87,1.06)	0.490	0.90 (0.81,0.90)	0.040
Beta blocking agent	15 021	0.89 (0.89,0.90)	0.83 (0.82,0.84)	0.80 (0.79,0.80)	0.76 (0.75,0.77)	0.74 (0.70,0.78)	0.001	0.81 (0.76,0.80)	0.001
Statin prophylaxis	17 691	0.88 (0.88,0.89)	0.82 (0.81,0.82)	0.78 (0.78,0.79)	0.75 (0.74,0.75)	0.80 (0.74,0.85)	0.001	0.95 (0.88,0.90)	0.300
Angiotensin converting enzyme, Angiotensin II receptor antagonist	11 752	0.88 (0.88,0.89)	0.82 (0.81,0.82)	0.78 (0.77,0.79)	0.75 (0.74,0.75)	0.92 (0.89,0.98)	0.005	0.98 (0.92,1.04)	0.570

^a^ Adjusted for demographic variables (gender, education, medical history of coronary heart disease, hypertension, diabetes and Hyperlipidemia, PCI/CABG, treatment, smoking), pre-heart attack symptoms, arrhythmia, complications and location of MI.

 Adjusted cox model demonstrated that the recipients of the group 2 drugs (Anticoagulants) had a significantly higher risk of death compared to those who did not receive this group (HR = 1.13 CI: 1.06-1.9) (regardless of the use of other drug groups or not). The patients who received drug groups 3, 5, and 6 had 36%, 10%, and 19% lower rates of death compared to their peers who did not receive these medications. Since most patients received combination group therapy, we analyzed the effect of the combination of drug groups in Table 4. As displayed in Table 4, the highest survival rate pertained to the recipients of medication groups (2, 3, 6, 8), (2, 4, 5), (2, 3, 6), and (1, 3, 4, 6). The lowest seven-year survival rate was in the recipients of medication groups (1), (3,6), (1,2,3,7), and (2,3,4,8). The survival rate in some was even lower than in the group who did not receive any medication.

**Table 4:** Survival rates by drug overlap at discharge

**Received drug groups**	**N**	**Survival Rate (95%CI)**				**Crude hazard ratio**		**Adjusted hazard ratio **^a^	
		**1-year **	**3-year **	**5-year **	**7-year **	**HR (95% CI)**	***P*** ** value**	**HR (95% CI)**	***P *** **value**
No received drugs	817	0.74 (0.71,0.77)	0.69 (0.66,0.72)	0.67 (0.64,0.70)	0.64 (0.61,0.67)	Ref.		Ref.	
Group 3	65	0.90 (0.80,0.95)	0.84 (0.73,0.91)	0.83 (0.71,0.90)	0.79 (0.67,0.87)	0.61 (0.35,1.05)	0.070	0.58 (0.34,1.00)	0.050
Groups 3,7	126	0.81 (0.73,0.87)	0.76 (0.68,0.83)	0.75 (0.66,0.81)	0.72 (0.63,0.79)	0.65 (0.46,0.93)	0.020	0.70 (0.49,1.00)	0.050
Groups 3,7, 8	85	0.84 (0.75,0.90)	0.74 (0.63,0.82)	0.69 (0.58,0.78)	0.68 (0.56,0.77)	0.86 (0.58,1.26)	0.450	0.80 (0.54,1.18)	0.260
Groups 3, 6	39	0.79 (0.63,0.89)	0.69 (0.52,0.81)	0.69 (0.52,0.81)	0.63 (0.46,0.86)	0.87 (0.51,1.49)	0.630	0.89 (0.52,1.52)	0.670
Groups 3, 6, 8	48	0.87 (0.74,0.94)	0.83 (0.69,0.91)	0.81 (0.67,0.89)	0.74 (0.59,0.84)	0.77 (0.43,1.38)	0.380	0.73 (0.41,1.30)	0.290
Groups 3, 6, 7	157	0.89 (0.83,0.93)	0.85 (0.78,0.90)	0.82 (0.75,0.87)	0.78 (0.71,0.84)	0.53 (0.37,0.76)	0.001	0.49 (0.34,0.71)	0.001
Groups 3, 6, 7, 8	284	0.90 (0.86,0.93)	0.84 (0.79,0.88)	0.79 (0.74,0.84)	0.77 (0.71,0.81)	0.61 (0.47,0.80)	0.001	0.62 (0.47,0.81)	0.001
Groups 3, 4	152	0.88 (0.82,0.92)	0.78 (0.70,0.84)	0.72 (0.65,0.79)	0.67 (0.59,0.74)	0.85 (0.63,1.16)	0.320	0.80 (0.59,1.09)	0.160
Groups 3, 4, 8	73	0.84 (0.74,0.91)	0.78 (0.66,0.85)	0.73 (0.62,0.82)	0.71 (0.59,0.80)	0.82 (0.53,1.28)	0.400	0.71 (0.45,1.13)	0.150
Groups 3, 6, 7	763	0.87 (0.84,0.89)	0.78 (0.75,0.81)	0.73 (0.70,0.76)	0.69 (0.66,0.72)	0.76 (0.64,0.90)	0.001	0.62 (0.52,0.73)	0.001
Groups 3,4, 7, 8	451	0.86 (0.83,0.89)	0.79 (0.75,0.83)	0.75 (0.71,0.79)	0.71 (0.67,0.75)	0.63 (0.51,0.78)	0.001	0.55 (0.45,0.68)	0.001
Groups 3, 4, 6	289	0.91 (0.87,0.94)	0.86 (0.82,0.90)	0.84 (0.79,0.87)	0.81 (0.76,0.85)	0.44 (0.33,0.59)	0.001	0.41 (0.31,0.55)	0.001
Groups 3, 4, 6, 8	183	0.91 (0.86,0.94)	0.86 (0.80,0.90)	0.81 (0.74,0.86)	0.77 (0.71,0.83)	0.55 (0.39,0.76)	0.001	0.52 (0.37,0.72)	0.001
Groups 3, 4, 6, 7	1087	0.91 (0.89,0.92)	0.84 (0.81,0.86)	0.80 (0.78,0.82)	0.77 (0.74,0.79)	0.53 (0.45,0.63)	0.001	0.50 (0.42,0.59)	0.001
Groups 3, 4, 6, 7,8	1930	0.92 (0.90,0.93)	0.86 (0.85,0.88)	0.83 (0.81,0.85)	0.79 (0.77,0.81)	0.47 (0.40,0.55)	0.001	0.43 (0.37,0.51)	0.001
Groups 3, 4, 5, 7	54	0.79 (0.66,0.88)	0.74 (0.60,0.83)	_	0.69 (0.55,0.80)	0.65 (0.39,1.09)	0.100	0.67 (0.40,1.11)	0.120
Groups 3, 4, 5, 7, 8	48	0.89 (0.76,0.95)	0.81 (0.67,0.89)	0.72 (0.57,0.83)	0.66 (0.50,0.77)	0.66 (0.40,1.10)	0.110	0.50 (0.30,0.83)	0.001
Groups 3, 4, 5, 6, 7	109	0.93 (0.87,0.96)	0.82 (0.74,0.88)	0.80 (0.71,0.86)	0.79 (0.70,0.86)	0.44 (0.28,0.68)	0.001	0.43 (0.27,0.66)	0.001
Groups 3, 4, 5, 7, 8	127	0.90 (0.83,0.94)	0.86 (0.79,0.91)	0.83 (0.75,0.88)	0.77 (0.68,0.83)	0.45 (0.30,0.67)	0.001	0.37 (0.25,0.55)	0.001
Groups 2, 3, 7	136	0.84 (0.77,0.89)	0.79 (0.71,0.85)	0.77 (0.69,0.83)	0.73 (0.64,0.79)	0.61 (0.43,0.87)	0.001	0.63 (0.44,0.89)	0.010
Groups 2, 3, 7, 8	107	0.87 (0.80,0.92)	0.75 (0.66,0.82)	0.73 (0.64,0.81)	0.69 (0.59,0.77)	0.70 (0.48,1.01)	0.060	0.61 (0.42,0.88)	0.010
Groups 2, 3, 6	49	0.95 (0.84,0.98)	0.91 (0.79,0.96)	0.87 (0.74,0.94)	0.85 (0.72,0.92)	0.47 (0.23,0.96)	0.030	0.37 (0.18,0.76)	0.001
Groups 2, 3, 6, 8	50	0.92 (0.80,0.96)	0.88 (0.75,0.94)	0.86 (0.72,0.93)	0.86 (0.72,0.93)	0.32 (0.15,0.67)	0.010	0.27 (0.12,0.57)	0.001
Groups 2, 3, 6, 7	157	0.87 (0.81,0.92)	0.80 (0.73,0.85)	0.77 (0.70,0.83)	0.73 (0.65,0.79)	0.70 (0.50,0.97)	0.030	0.70 (0.50,0.97)	0.030
Groups 2, 3, 6, 7, 8	318	0.89 (0.85,0.92)	0.83 (0.78,0.86)	0.78 (0.73,0.82)	0.75 (0.70,0.79)	0.64 (0.50,0.82)	0.001	0.56 (0.43,0.72)	0.001
Groups 2, 3, 4	206	0.83 (0.78,0.88)	0.77 (0.70,0.82)	0.75 (0.68,0.80)	0.72 (0.65,0.78)	0.67 (0.50,0.89)	0.001	0.61 (0.46,0.81)	0.001
Groups 2, 3, 4, 8	76	0.81 (0.70,0.88)	0.75 (0.63,0.83)	0.69 (0.58,0.78)	0.65 (0.53,0.75)	0.98 (0.66,1.47)	0.950	0.86 (0.58,1.30)	0.490
Groups 2, 3, 4, 7	777	0.86 (0.83,0.88)	0.79 (0.76,0.81)	0.75 (0.72,0.78)	0.73 (0.70,0.76)	0.70 (0.59,0.84)	0.001	0.69 (0.58,0.83)	0.001
Groups 2, 3, 4, 7, 8	772	0.85 (0.82,0.87)	0.76 (0.73,0.79)	0.71 (0.68,0.74)	0.67 (0.64,0.71)	0.75 (0.63,0.88)	0.001	0.67 (0.56,0.79)	0.001
Groups 2, 3, 6	331	0.88 (0.84,0.91)	0.80 (0.76,0.84)	0.77 (0.72,0.81)	0.73 (0.67,0.77)	0.65 (0.51,0.82)	0.001	0.59 (0.46,0.75)	0.001
Groups 2, 3, 4, 6, 8	250	0.90 (0.86,0.93)	0.79 (0.74,0.84)	0.77 (0.71,0.81)	0.73 (0.67,0.78)	0.65 (0.50,0.85)	0.001	0.56 (0.43,0.73)	0.001
Groups 2, 3, 4, 6, 7	2091	0.90 (0.89,0.91)	0.84 (0.82,0.85)	0.81 (0.79,0.82)	0.77 (0.75,0.79)	0.56 (0.48,0.65)	0.001	0.53 (0.46,0.62)	0.001
Groups 2, 3, 4, 6, 7, 8	4486	0.88 (0.88,0.89)	0.82 (0.81,0.83)	0.79 (0.77,0.80)	0.75 (0.74,0.76)	0.61 (0.54,0.70)	0.001	0.55 (0.49,0.63)	0.001
Groups 2, 3, 4, 5, 7	92	0.91 (0.83,0.95)	0.88 (0.79,0.93)	0.83 (0.74,0.89)	0.77 (0.67,0.85)	0.54 (0.34,0.86)	0.010	0.45 (0.28,0.70)	0.001
Groups 2, 3, 4, 5, 7, 8	110	0.82 (0.74,0.88)	0.79 (0.71,0.86)	0.72 (0.63,0.79)	0.67 (0.58,0.75)	0.71 (0.50,1.01)	0.060	0.59 (0.41,0.84)	0.001
Groups 2, 3, 4, 5, 6	36	0.88 (0.73,0.95)	0.80 (0.63,0.90)	0.71 (0.54,0.83)	0.69 (0.51,0.81)	0.64 (0.35,1.17)	0.150	0.68 (0.37,1.25)	0.210
Groups 2, 3, 4, 5, 6, 7	161	0.91 (0.85,0.94)	0.85 (0.79,0.90)	0.81 (0.75,0.87)	0.78 (0.71,0.84)	0.47 (0.33,0.67)	0.001	0.44 (0.31,0.61)	0.001
Groups 2, 3, 4, 5, 6, 7, 8	343	0.86 (0.82,0.90)	0.81 (0.76,0.85)	0.78 (0.73,0.82)	0.74 (0.69,0.78)	0.61 (0.48,0.77)	0.001	0.48 (0.37,0.61)	0.001
Group 1	158	0.72 (0.64,0.78)	0.66 (0.58,0.73)	0.63 (0.55,0.70)	0.59 (0.50,0.66)	1.21 (0.92,1.59)	0.150	1.21 (0.92,1.58)	0.160
Groups 1, 3, 6, 7, 8	46	0.86 (0.73,0.93)	0.80 (0.65,0.89)	0.76 (0.60,0.85)	0.73 (0.57,0.83)	0.65 (0.36,1.15)	0.140	0.61 (0.34,1.09)	0.090
Groups 1, 3, 4, 7	139	0.88 (0.81,0.92)	0.79 (0.71,0.84)	0.74 (0.66,0.81)	0.71 (0.63,0.78)	0.70 (0.50,0.97)	0.030	0.53 (0.38,0.75)	0.001
Groups 1, 3, 4, 7, 8	86	0.84 (0.75,0.90)	0.76 (0.66,0.84)	0.73 (0.62,0.81)	0.73 (0.62,0.81)	0.65 (0.43,1.00)	0.050	0.63 (0.41,0.97)	0.030
Groups 1, 3, 4, 6	36	0.97 (0.81,0.99)	0.88 (0.72,0.95)	0.88 (0.72,0.95)	0.85 (0.68,0.93)	0.34 (0.15,0.77)	0.010	0.40 (0.18,0.91)	0.020
Groups 1, 3, 4, 6, 7	220	0.90 (0.85,0.93)	0.81 (0.75,0.86)	0.79 (0.73,0.84)	0.73 (0.67,0.78)	0.66 (0.50,0.88)	0.001	0.63 (0.48,0.84)	0.001
Groups 1, 3, 4, 6, 7, 8	346	0.90 (0.86,0.93)	0.81 (0.77,0.85)	0.80 (0.76,0.84)	0.78 (0.74,0.82)	0.52 (0.41,0.68)	0.001	0.46 (0.36,0.60)	0.001
Groups 1, 2, 3, 7	34	0.79 (0.61,0.89)	_	_	0.64 (0.45,0.77)	0.90 (0.50,1.60)	0.720	077 (0.43,1.38)	0.390
Groups 1, 2, 3, 6, 7, 8	62	0.95 (0.85,0.98)	0.85 (0.73,0.91)	0.83 (0.71,0.90)	0.76 (0.63,0.85)	0.53 (0.31,0.90)	0.010	0.49 (0.29,0.83)	0.001
Groups 1, 2, 3, 4	33	0.87 (0.70,0.95)	_	0.81 (0.63,0.91)	0.78 (0.60,0.89)	0.49 (0.23,1.04)	0.060	0.41 (0.19,0.88)	0.020
Groups 1, 2, 3, 4, 7	139	0.86 (0.79,0.91)	0.82 (0.75,0.88)	0.76 (0.68,0.82)	0.73 (0.65,0.80)	0.60 (0.42,0.84)	0.001	0.59 (0.42,0.83)	0.001
Groups 1, 2, 3, 4, 7, 8	135	0.82 (0.74,0.87)	0.76 (0.68,0.82)	0.75 (0.67,0.81)	0.69 (0.61,0.76)	0.74 (0.53,1.03)	0.070	0.61 (0.43,0.85)	0.001
Groups 1, 2, 3, 4, 6	60	0.91 (0.81,0.96)	0.85 (0.73,0.91)	0.81 (0.69,0.89)	0.76 (0.63,0.86)	0.63 (0.37,1.08)	0.090	0.66 (0.39,1.14)	0.140
Groups 1, 2, 3, 4, 6, 7	373	0.87 (0.83,0.90)	0.82 (0.78,0.86)	0.79 (0.75,0.83)	0.76 (0.72,0.80)	0.65 (0.51,0.83)	0.001	0.63 (0.50,0.81)	0.001
Groups 1, 2, 3, 4, 6, 7, 8	761	0.87 (0.85,0.90)	0.81 (0.79,0.84)	0.79 (0.76,0.81)	0.75 (0.72,0.78)	0.59 (0.49,0.71)	0.001	0.54 (0.45,0.65)	0.001
All groups drug	50	0.88 (0.75,0.94)	0.80 (0.66,0.88)	0.70 (0.55,0.80)	0.65 (0.50,0.77)	0.80 (0.49,1.31)	0.380	0.68 (0.42,1.12)	0.130

^a^ adjusted for demographic variables (gender, education, medical history of coronary heart disease, hypertension, diabetes and Hyperlipidemia, PCI/CABG treatment, smoking), pre-heart attack symptoms, arrhythmia, complications and location of MI.

 Regarding the combined reception of the medication groups, the most significant reduction in the risk of death for the combined reception of the medication groups was for the drug groups 2, 3, 6, and 8 (HR = 0.27), while the lowest reduction in the risk was for the drug group 3 and 6 (HR = 0.89). The group that did not receive any medication was considered a reference. The effect of other variables is also adjusted in the model. Table 5 illustrates a positive past medical history of coronary artery diseases, hypertension (HR = 1.7, CI: 1.10-1.24), smoking (HR = 1.03, CI: 1.01-1.06), arrhythmia (HR = 1.59, CI: 1.48-1.17), and receiving PCI/CABG treatments (HR = 1.07, CI: 1.02-1.12) significantly increased the risk of death. On the other hand, the history of Hyperlipidemia (HR = 0.90, CI: 0.84-0.97), higher levels of education (HR = 0.62, CI: 0.57-0.65), and post-MI complications (HR = 0.91, CI: 0.87-0.95) significantly reduced the risk of death. Furthermore, MI at the anterior wall, inferior wall, and other sites together caused a significant increase in hazard ratios.

**Table 5:** Factors affecting survival in patients with acute myocardial infarction

**Variables**	**Crude hazard ratio (95% CI)**	***P*** ** value**	**Adjusted hazard ratio (95% CI)** ^a^	***P*** ** value**
Age	1.22 (1.21, 1.23)	0.001	1.12 (1.10, 1.14)	0.001
Gender (Female to male)	1.31 (1.23, 1.39)	0.001	1.05 (0.98, 1.12)	0.100
Education level (literate to illiterate)	0.61 (0.57, 0.65)	0.001	0.62 (0.57, 0.65)	0.001
Coronary artery diseases	1.61 (1.52, 1.71)	0.001	1.46 (1.37, 1.56)	0.001
Hypertension	1.38 (1.31, 1.46)	0.001	1.17 (1.10, 1.24)	0.001
Diabetes	1.57 (1.48, 1.67)	0.001	1.51 (1.39, 1.63)	0.010
Interaction with time (diabetes)	-	-	0.97 (0.94, 1.00)	0.060
Hyperlipidemia	0.93 (0.86, 1.00)	0.060	0.90 (0.84, 0.97)	0.001
Interaction with time (PCI/CABG)	-	-	1.07 (1.02, 1.12)	0.001
PCI/CABG treatments	1.24 (1.11, 1.37)	0.001	0.92 (0.80, 1.70)	0.320
Interaction with time (cigarette smoking)	-	-	1.03 (1.01, 1.06)	0.010
Cigarette smoking	0.94 (0.88, 1,00)	0.070	0.95 (0.86, 1.03)	0.190
Clinical symptoms	1.05 (0.99, 1.12)	0.070	1.04 (0.98, 1.11)	0.180
Arrhythmia	1.67 (1.59, 1.80)	0.001	1.59 (1.48, 1.17)	0.001
Interaction with time (complications post-MI)	-	-	0.91 (0.87, 0.95)	0.001
Complications post-MI	1.47 (1.35, 1.60)	0.001	1.65 (1.48, 1.84)	0.001
Acute transmural myocardial infarction of another site	1.15 (0.96, 1.37)	0.070	1.21 (1.10, 1.47)	0.040
Acute transmural myocardial of the inferior wall	1.01 (0.95, 1.07)	0.360	1.07 (0.98, 1.17)	0.100
Acute transmural myocardial infarction of the anterior wall	1.02 (0.92, 1.06)	0.600	1.03 (1.01, 1.05)	0.010
Acute myocardial infarction of unspecified site	0.92 (0.76, 1.05)	0.340	1.06 (0.99, 1.14)	0.050
Acute transmural myocardial infarction of the inferior wall and anterior wall	1.10 (0.89, 1.27)	0.300	1.12 (0.92, 1.35)	0.230
Acute transmural myocardial infarction of the anterior wall and other sites	1.06 (0.89, 1.27)	0.350	0.84 (0.63, 1.10)	0.210
Acute transmural myocardial infarction of the inferior wall and other sites	1.07 (0.91, 1.26)	0.230	1.15 (0.96, 1.37)	0.100
Myocardial infarction of non-ST elevation with location	1.03 (0.95, 1.12)	0.240	1.06 (0.95, 1.18)	0.240
Acute transmural myocardial infarction of the anterior wall, inferior wall, and other sites	1.37 (1.00, 1.87)	0.030	1.46 (1.06, 2.01)	0.020

^a^ Drug compounds received at the time of discharge are also included in the model.

## Discussion

 Survival is the main interesting outcome after acute MI. By calculating the survival rate and effective factors influencing the survival of patients with MI, it is possible to provide optimum services for patients, as well as special measures to control and reduce the mortality rate due to acute MI, and prolong the life and survival of these patients.

 In this study, survival rates at all time periods were significantly lower in women than in men. In the same context, Bucholz et al. investigated gender differences in long-term survival. In most studies, the survival rate was lower in women than in men; in general, most studies reported higher unadjusted mortality in women than in men in 5 and 10 years after acute MI. Nonetheless, many differences in mortality decreased after adjustment for age.^13^ In the study by Johnston et al., gender differences in the assessment of five-year survival of patients, demonstrated that women were more likely to die than men during the first year after MI (6.2% versus 4.1%) and consistent with the results of the present study, women had less survival rate than men.^14^

 The survival rate of patients was over the period of 1-year (88 %), 3-year (81 %), 5-year (78 %), and 7-year (74 %). MI still has high mortality rates, and most deaths occur before reaching the hospital. At least 5%-10% of survivors die in the first 12 months of their MI, and nearly 50% need to be rehospitalized in the same year. The prognosis is dependent on the amount of heart muscle damage. Good results are yielded in patients undergoing the thrombolytic perifusion treatment in the first 30 min after the arrival and receiving PCI operation in the first 90 min. In the study by Mosa Farkhani et al, the 1-year survival rate was 80 %, and survival was estimated to be 64 % in the total period of five years.^15^

 In the study by Malik et al in Pakistan, the 1-year survival rate was 66.7%,^16^ less than the observed survival rate in our study, which could be due to the higher coverage of therapeutic actions and medical facilities in Iran. In agreement with the results of the present research, in a study by Nadlacki et al in Australia, the rate of 1-year survival was 85.9%, 3-year survival was 68.6%, and the total rate of the 7-year survival period was calculated at 62.3%.^11^ The reported 7-year survival rate in a study in Sweden (2013-2014) was about 70% for patients with ST-segment elevation myocardial infarction (STEMI) and 60% for patients with non-ST-elevation myocardial infarction (NSTEMI**)**.^17,18^ The survival rate in the current study was higher. In addition to the antithrombotic therapies, β blockers, ACE inhibitors, and aldosterone antagonists have been shown to improve long-term outcomes in selected patients after MI.^19^

 In the study by Safi et al, it was indicated that beta-blockers for suspected or diagnosed acute MI probably reduce the short-term risk of reinfarction, as well as the long-term risk of all-cause mortality and cardiovascular mortality. Nevertheless, it is most likely that beta-blockers have little or no effect on the short-term risk of all-cause mortality and cardiovascular mortality.^20^ The results were consistent with the protective effect of patients’ risk of death associated with the use of beta-blockers.

 Diuretics are effective in the reduction of cardiovascular events in patients with hypertension; moreover, they are more effective than β-blockers and ACE inhibitors in reducing stroke. In the present study, the effect of diuretics was associated with a decrease in patient survival; nonetheless, it was not statistically significant. Most recent guidelines continue to recommend thiazide-related diuretics as first-line agents for all patients with hypertension.^21^

 The results of a study by Ann et al indicated that ACE inhibitors treatment in patients with AMI and concomitant PCI demonstrated a significant reduction in all-cause mortality compared to angiotensin receptor blockers treatment. In this study, a protective effect was observed in all groups that received one of these two drugs in all combination therapies with other prescribed drugs.^22^ The role of aspirin in the primary prevention of CVD is controversial. Early trials evaluating aspirin for primary prevention suggested reductions in MI and stroke (although not mortality), as well as an increased risk of bleeding.^23^

 In the largest primary prevention aspirin trial in 2018 that examined the use of aspirin among older patients (aged ≥ 65 years), no difference was seen between the two groups (HR 0.95, 95% CI 0·83-1.08) in cardiovascular events, including fatal and non-fatal MI and stroke. It was in contrast with the protective effect observed in our study.^24^ Taking antiplatelet drugs together with aspirin prevents the accumulation of platelets inside the arteries and helps to reduce the risk of re-clogging the arteries, as well as the occurrence of a heart attack.

 In a meta-analysis conducted by Chiarito et al, the findings supported differential treatment effects of anticoagulants, in addition to antiplatelets, according to clinical presentation. In patients with acute coronary syndrome, the risk-benefit profile of anticoagulants appears unfavorable. Conversely, anticoagulants, in addition to antiplatelets, might represent an attractive option for patients with MI. In our study, the single use of group1 in medication compounds was associated with a higher risk of mortality, although it was not statistically significant (Table 4).^25^

 In a meta-analysis conducted by Chopra et al, perioperative statin treatment in patients reduced atrial fibrillation, MI, and duration of hospital stay. The wider use of statins to improve cardiac outcomes in patients undergoing high-risk procedures seems warranted. The present study also confirmed the results.^26^ In the study by Pedrinelli et al, about 72% of the selected samples of MI had hypertension.^27^ In fact, most studies have pointed to the relationship between hypertension and MI. In the present research, hypertension was also a risk factor for mortality.

 Smoking is considered a strong risk factor for MI, premature atherosclerosis, and sudden cardiac arrest. Smoking leads to premature death by the diagnosis of STEMI in patients, especially in healthier patients.^28,29^ Gao et al reported that smoking was positively associated with the risk of developing respiratory diseases, hypertension, and MI during the life period; moreover, consistent with the results of our study, this risk increases with age.^30^ Although smoking is known as a risk factor for health, some studies have recorded contradictory findings on hypertension and MI. For instance, some researchers have reported lower blood pressure levels among smokers compared to former smokers and reported an increase in blood pressure after quitting smoking.^31,32^ In our study, the association between smoking and mortality was statistically significant.

 In line with the results of the present study, Quinones et al analyses showed strong protective effects only among men and women younger than 60 years diagnosed with hyperlipidemia.^33^ Among the notable limitations of this study, we can refer to the lack of access to care and adherence to the treatment in the follow-up period of the patients with acute MI and not registering of deaths due to MI that happened before reaching the hospital, having no access to secondary infarctions records in patients, and non-access to variables that change over time and affect the survival of patients.

## Conclusion

 As evidenced by the results of this study, different combinations of prescribed medication drugs had protective effects on long-term mortality compared to the group without any drug. Nonetheless, according to the drugs in each combination therapy, this protective effect ranged from HR = 0.27 to HR = 0.89. Further studies are recommended to compare the long-term effects of different drug combinations and also consider adherence to treatment in evaluating the impact of these combination therapies.

**Highlights**

- This study provides estimates of long-term survival rates for people with a diagnosis of MI.
- The pharmacological treatment of patients with MI can play a preventive role against death. Most CVDs are preventable.
- The rate of death can be reduced due to such risk factors as smoking, hypertension, diabetes, and hyperlipidemia.

## Acknowledgments

 We would like to thank the Ministry of Health and Medical Education of Iran which participated in this project and provided us with its services.

## Conflicts of interest

 The authors declare that they have no conflict of interest.
